# 

**Published:** 1899-11

**Authors:** 


### Society Notes.

### Western Surgical and Gynecological Association.

The ninth annual meeting of the Western Surgical and Gynecological Association will be held at Des Moines, Iowa, December 27 and 28, 1899. Surgeons and gynecologists of the great West are cordially invited to affiliate themselves with this Association. The secretary will be glad to send application-blanks on request. Titles of papers should be sent to the secretary as soon as convenient, but not later than November 20, to insure a place on the program.

H. C. CROWELL, President,
GEORGE H. SIMMONS, Kansas City, Mo.
Sec'y and Treas., 61 Market St., Chicago.

### Rrazos Valley Medical Association—Eighth Semi-Annual Meeting on Tuesday and Wednesday, November 14 and 15, 1899, at Hearne.

DEAR DOCTOR: It is with much pleasure that I send to you this, our programe, of the eighth semi-annual meeting of the Brazos Valley Medical Association, which convenes at Hearne, Texas, on Tuesday and Wednesday, November 14 and 15, 1899.

All charter members especially should feel it their duty to attend the fourth anniversary of our Association, for at Hearne we began

*The address of Dr. Lankford, the retiring President, and a paper by Dr, J. H. Reuss of Cuero, were received too late for this issue, but will be publishe d in the next.—ED.

life, and from the impetus there given, we have become one of the leading medical societies in the South.

New members and visitors should also be with us. We all need a rest from business cares and the labor of our profession, and there is no more suitable time nor place to renew old friendship and give new enthusiasm to our Association.

We respectfully beg that you bring some clinical subjects with you, and also, come prepared to report whatever interesting cases you may have had in your practice.

We desire to make this the most practical meeting in the history of our Association, and I earnestly entreat each member to do his part towards making it a success.

The local physicians and citizens of Hearne may be relied on to make our stay with them pleasant and enjoyable. I am,

Fraternally yours,

I. P. SESSIONS, President.

The officers of the Association are I. P. Sessions, M. D., president, Rocdale; W. B. Briggs, M. D., secretary, Easterly.

[Program received too late to be given in full in this issue. It embraces seven papers in Section Obstetrics, etc.; eight in General Medicine, and nine in Surgery and Gynecology.—ED.]

